# Nutritional application of olive pomace in dairy animals: chemical composition, implications for milk quality and yield, nutrient digestibility, economics, and limitations

**DOI:** 10.5194/aab-68-731-2025

**Published:** 2025-12-11

**Authors:** Rana Muhammad Bilal, Hassan A. Rudayni, Faiz Ul Hassan, Muhammad Uzair Akhtar, Kashif Prince, Abdullah S. Alawam, Ahmed A. Allam, Mayada Ragab Farag, Asmaa F. Khafaga, Ayman E. Taha, Mahmoud Alagawany

**Affiliations:** 1 Department of Animal Nutrition, Cholistan University of Veterinary and Animal Sciences, Near DHA, Bahawalpur 63100, Pakistan; 2 Department of Biology, College of Science, Imam Mohammad Ibn Saud Islamic University (IMSIU), Riyadh 11623, Saudi Arabia; 3 Department of Breeding and Genetics, Cholistan University of Veterinary and Animal Sciences, Near DHA, Bahawalpur 63100, Pakistan; 4 Department of Medicine, Cholistan University of Veterinary and Animal Sciences, Bahawalpur 63100, Pakistan; 5 Forensic Medicine and Toxicology Department, Veterinary Medicine Faculty, Zagazig University, Zagazig 44519, Egypt; 6 Department of Pathology, College of Veterinary Medicine, King Faisal University, Al-Ahsa 31982, Saudi Arabia; 7 Department of Public Health, College of Veterinary Medicine, King Faisal University, Al-Ahsa 31982, Saudi Arabia; 8 Poultry Department, Agriculture Faculty, Zagazig University, Zagazig 44511, Egypt

## Abstract

The olive oil industry produces large volumes of by-products which, if not properly managed, can negatively impact water resources, aquatic ecosystems, soil quality, and the atmosphere. The extraction process generates several by-products that have previously been regarded as waste. Utilizing these residues as alternative feed ingredients aligns with the principles of a circular economy, making the agri-food system more sustainable, conserving natural resources, and reducing the environmental footprint of livestock production. Therefore, the proper use of industrial by-products is of great importance and requires continuous attention. Olive pomace, a major by-product of olive oil extraction, can be used in animal feed as an inexpensive yet nutritious residue. It consists mainly of pasty pulp, kernel, skin, and vegetation water. Once dried, it becomes a stable by-product suitable for feed use. Olive by-products can be incorporated into animal diets in several forms, such as fresh, ensiled, or dried material, or as components of concentrate pellets and multi-nutrient feed blocks. Among these, dried olive pomace is considered one of the most practical and stable options. Olive pomace, as a by-product of the olive oil industry, is rich in phenolic compounds and beneficial fatty acids that play significant roles in animal health and productivity. Hence, it is crucial to evaluate its potential as a feed ingredient; however, current understanding of its specific effects on livestock production remains limited. This review focuses on the influence of olive pomace inclusion in animal diets on milk production, growth performance, nutrient digestibility, feed efficiency, and overall health status. In general, dietary olive pomace has shown satisfactory results, serving as a low-cost nutrient source that can enhance both the productivity and the quality of animal-derived products.

## 1 Introduction

In recent years, climate change, global economic shifts, and the growing demands of the food industry have significantly affected the availability and cost of conventional feed ingredients for livestock (Sevillano et al., 2025; Schú et al., 2025). Limited grazing land in many regions has intensified the challenge of maintaining cost-effective animal production systems (Sucu et al., 2018). Consequently, identifying sustainable, low-cost feed alternatives has become essential to ensure the economic and environmental viability of livestock farming. The utilization of agro-industrial by-products as feed ingredients aligns with the principles of a circular economy, contributing to the reduction of waste, conservation of resources, and minimization of environmental impacts (Mathabela et al., 2025).

The olive oil industry, particularly in Mediterranean countries such as Greece, Italy, and Spain, generates large volumes of by-products, with approximately 800 kg of olive pomace and 200 kg of oil produced per 1000 kg of olive fruits (Castellani et al., 2017; Tzamaloukas et al., 2021; Obeidat and Kridli, 2021). Olive pomace, the solid residue remaining after oil extraction, is composed mainly of pulp, skin, kernel, and vegetation water (Bilal et al., 2021). Depending on the extraction method, it can be used fresh, ensiled, or dried as a feed ingredient (Nanas et al., 2025). Dried olive pomace (DOP) is considered the most stable and manageable form, providing a valuable source of nutrients and bioactive compounds such as phenolics and fatty acids beneficial for animal health and production (Benincasa et al., 2021).

Previous studies have explored the inclusion of olive pomace in various livestock diets with promising results. It has been used untreated, alkali-treated, or as silage without negative effects on milk yield or growth performance (Omar et al., 2012; Castellani et al., 2017). In ruminants such as dairy cows, ewes, goats, and camels, the dietary addition of olive pomace generally did not impair milk composition or production (Chaves et al., 2021; Neofytou et al., 2020; Aljamal et al., 2021; Faye et al., 2013). Some studies even reported improved dry matter (DM) intake and carcass quality in lambs (Abid et al., 2020; Awawdeh and Obeidat, 2013; Hamdi et al., 2016). However, discrepancies exist, as other investigations found reduced nutrient digestibility, including moisture-free content (DM) and fibre fractions such as structural fibre (NDF) and resistant fibre (ADF), as well as the nitrogen balance at higher inclusion levels (Owaimer et al., 2004; García et al., 2003; Tufarelli et al., 2013; Abbeddou et al., 2011).

Despite these variations, the inclusion of olive pomace in ruminant diets has been associated with improved fatty acid profiles in milk and meat, particularly due to its high oleic acid content (Castellani et al., 2017; Scicutella et al., 2023; Amato et al., 2024). Moreover, feeding olive pomace offers potential economic benefits by lowering feed costs and contributing to sustainable waste management (Mele et al., 2014; Mohammadabadi et al., 2025). The growing interest in high-quality, environmentally friendly animal products has further encouraged research into olive by-products as functional feed ingredients. However, despite growing research interest, the current literature lacks a consolidated and critical synthesis of how olive pomace bioactives influence health and productivity parameters in dairy species. Considering the available but fragmented evidence, this review aims to provide an updated overview of the chemical composition of olive pomace and to summarize its effects on milk yield, milk quality, nutrient digestibility, economic feasibility, and nutritional limitations in dairy animals.

## 2 Chemical composition of olive pomace

To determine whether alternative feed sources are suitable, it is essential to understand their nutrient content. Because these by-products can have a variety of nutrients, it is necessary to analyse factors like including moisture-free content, protein levels, structural fibres, resistant fibres, and fat content before adding them in animal diets (Nunes et al., 2021; Carboni et al., 2025). In areas like southern Punjab, limited rainfall and water scarcity mean that pastures and range-based forages are not readily available. As a result, many livestock rely on wheat and barley straws as a major feed. However, these straws not only provide low nutritional value and poor digestibility but are also not cost-effective for feeding (Obeidat et al., 2019).

**Figure 1:**
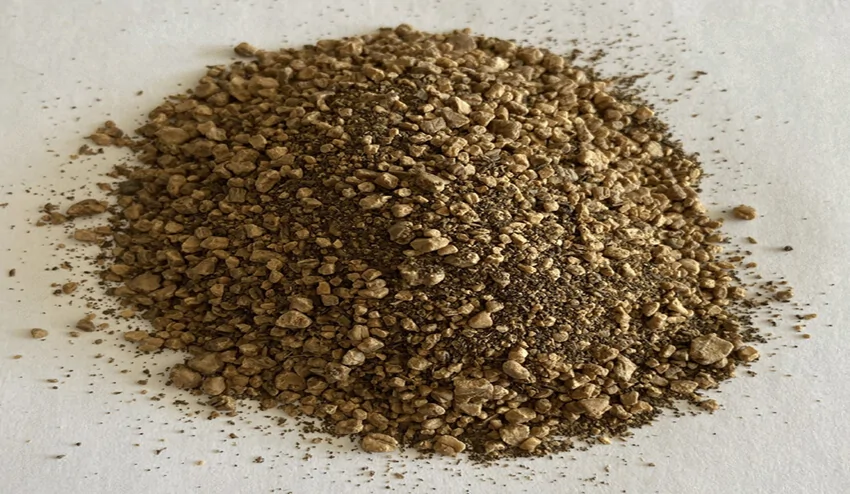
Olive pomace.

The chemical composition of olive pomace (Fig. 1) depends on several factors, such as the time of year, how the oil is extracted, how ripe the olives are, and where they are grown (area). On a DM basis, it generally contains about 51 % DM, 95 % organic matter (OM), 6.5 % crude protein (CP), 54 % neutral detergent fibre (NDF), 37 % acid detergent fibre (ADF), and 22 % ether extract (EE) (Obeidat and Kridli, 2021). Olive pomace is abundant in β carotene, comprising carotenoids and lutein, which enhance the yellow colour of fat in lamb meat (Ghanbari et al., 2012).

Levels of polyphenol compounds in olive pomace are largely dependent on the methods of extraction employed. Crushing, malaxation, and drying are the most important steps in the extraction process because they lower or stop the activity of bioactive chemicals in olive pomace (de Oliveira et al., 2021). Bioactive components in olive pomace include oleuropeoside molecules (verbascoside and oleuropein), flavanols (catechins), flavonoids (luteolin-7-glucoside, luteolin, diosmetin, diosmetin-7-glucoside, apegenin-7-glucoside, and rutin), simple phenolic compounds (hydroxytyrosol, tyrosol, vanillic acid, vanillin, and caffeic acid), etc. (Ryan et al., 2002). Olive by-products provide a suitable background to use them in the nutrition and feed industry. Olive pomace is rich in oil residues and has a good fibre content, low CP, a lignocellulosic matrix, unsaturated fatty acids, saturated fatty acids, phenolic compounds, and tocopherols. All of these compounds are very good antioxidants (Benincasa et al., 2021). The nutrient composition for olive pomace has been reported in the literature, as presented in Table 1.

**Table 1:** Nutrient composition of olive pomace reported in the literature.

Literature	Chemical composition (%)^*^					
	DM	CP	EE	CF	Total ash	Energy, kcal kg^−1^
Martin Garcia et al. (2003)	87.10	7.88	0.13	–	–	4560 (GE)^*^
Molina-Alcaide and Yáñez-Ruiz (2008)	80.50	7.26	5.45	41.10	–	4804 (GE)^*^
Zarei et al. (2011)	87.00	10.20	–	24.00	–	–
Afsari et al. (2013)	93.00	6.10	7.60	48.20	7.40	–
Al-Harthi and Attia (2016)	–	5.20	11.80	14.10	20.40	1600 (ME)^*^
Abd El-Galil et al. (2017)	–	9.10	9.00	18.50	7.50	2463 (ME)^*^
Nasopoulou et al. (2018)	94.10	9.80	18.30	21.50	7.10	4400 (ME)^*^
El-Moneim and Sabic (2019)	87.20	9.70	10.70	20.00	8.00	–
Ibrahim et al. (2019)	90.01	10.70	12.00	24.00	7.50	3751 (ME)^*^
Rebollada-Merino et al. (2019)	87.80	6.40	3.00	27.70	7.70	–
Iannaccone et al. (2019)	67.20	7.80	15.50	–	–	–
Papadomichelakis et al. (2019)	94.50	8.60	17.50	27.50	–	2675 (ME)^*^
Pappas et al. (2019)	93.00	6.10	7.60	48.20	7.40	2675 (ME)^*^
Quero et al. (2022)	93.83	5.66	12.06	–	4.55	–
Abdelhamid et al. (2025)	66.22	9.43	9.53	31.49	3.16	–

^*^ DM – dry matter, CP – crude protein, EE – ether extract, CF – crude fibre, ME – metabolizable energy, GE – gross energy.

## 3 Effects of olive pomace on milk yield and milk composition

The potential improvement of milk composition in ruminants upon inclusion of olive pomace is often associated with the fatty acid profile of milk (Mohammadabadi et al., 2025). Olive pomace in animal feeding is a very healthy and sustainable alternative and can also decrease feeding costs and increase the quality of dairy products (Chiofalo et al., 2004). Molina-Alcaide and Yáñez-Ruiz (2008) have shown that low-level supplementation of olive pomace in small-ruminant feed is associated with increased milk quality and production and a higher nutritional composition of milk. Animal diet is the key reason behind the biohydrogenation (BH) in rumen, and thus the feed quality influences the ruminal fatty acid (FA) composition and thus accordingly also affects the quality and quantity of meat and milk. The substitution of olive pomace at 15 % in the feed significantly enhances the diet's EE to approximately 60 % (Hadjipanayiotou, 1999).

Sources of fatty acid in the diet are mainly detrimental to the composition of fatty acids in the milk. The primary fatty acid in olive pomace is oleic acid (C18:1 cis-9), making up the largest proportion. Palmitic acid (C16:0) and linoleic acid (C18:1 cis-9, cis-12) are also present in significant amounts, but the concentrations of stearic acid (C18:0) and linolenic acid (C18:3 cis-9, cis-12, cis-15) are much lower in dried olive pomace (Castellani et al., 2017).

Normally, adding polyphenols to ruminant feeds has been observed to reduce the BH of dietary polyunsaturated fatty acids (PUFAs) by influencing the microbial population, its varieties, and their activity. The presence of polyphenols boosts the flow of bioactive fatty acids in the duodenum-like vaccenic acid (trans-11 C18:1, VA), hence improving the dietary value of milk fat percentage. As olive pomace is rich in certain unsaturated fatty acids that are beneficial for health, like linoleic acid and oleic acid, it can influence the lipid composition of milk through higher energy provision and hence can positively impact milk and meat production. Olive pomace, being rich particularly in oleic acid (C18:1; a beneficial fatty acid), can ameliorate the oleic acid content in milk fat upon inclusion in ruminants' diet, while linoleic acid (C18:2; a polyunsaturated fatty acid) is not as abundant as oleic acid but can contribute to the overall fatty acid profile of milk and meat (Terramoccia et al., 2013; Zilio et al., 2014). However, olive pomace supplementation did not increase the milk yield in ruminants and its cost-effectiveness (Owaimer et al., 2004; Awawdeh and Obeidat, 2013).

According to research by Pallara et al. (2014), olive by-products have lower digestibility, protein availability, palatability, and microbial protein production, most probably because of their higher polyphenol contents, which can inhibit the extracellular enzymes that ruminal microflora secretes. Pallara et al. (2014) observed that feeding stoned olive cake in a ruminant diet led to a decrease in the biohydrogenation of C18 unsaturated FA in the rumen during in vitro studies, thereby reducing stearic acid levels while simultaneously increasing vaccenic acid concentrations. This may be linked to changes in microbial activities and populations, suppressing the bacteria producing the stearic acid. Moreover, when the two-stage olive cake was added, vaccenic acid production increased due to its richness in linoleic and oleic fatty acids. In goat milk, higher concentrations of total conjugated linoleic acid and rumenic acid were observed due to linoleic acid isomerization and vaccenic acid desaturation occurring in both the rumen and the mammary gland (Molina-Alcaide et al., 2010).

Saturated fatty acids (SFAs) like stearic acid (C18:0) and palmitic acid (C16:0) are common in the fats of many feed ingredients, including olive pomace. However, the presence of vaccenic acid (VA) in ruminant products is often reduced due to its conversion into stearic acid (C18:0) by rumen microbes. Pallara et al. (2014) conducted in vitro trials with olive pomace to examine its effects on rumen biohydrogenation and showed its great influence on the PUFAs, especially linoleic acid (LA; 18:2 cis9 cis12). Chiofalo et al. (2004) reported a notable rise in the UFA / SFA ratio along with a reduction in the atherogenic and thrombogenic indices of milk by feeding stoned olive cakes. Animals supplemented with olive pomace resulted in a decrease in the proportion of short- and medium-chain fatty acids along with an increase in oleic acid levels in milk (Abbeddou et al., 2011; Vargas-Bello-Pérez et al., 2013).

Many studies have reported inconsistent findings, which may be attributed to the varying inclusion levels of olive oil pomace (OOP) and its interactions with specific diets (Chiofalo et al., 2004; Molina-Alcaide et al., 2010; Abbeddou et al., 2011; Mohammadabadi et al., 2025). In the case of milk urea, olive pomace does not influence dietary protein metabolism in the same manner as tannins (Chilliard et al., 2007). The lower levels of short- and medium-chain fatty acids (SMCFAs) in milk fat from ruminants that were fed olive pomace are likely due to a decrease in the production of fat in the mammary gland (Min et al., 2003). Chilliard et al. (2007) found that animals that were fed olive pomace had higher oleic acid (OA) content in their milk fat, which is mainly produced by the mammary Δ 9-desaturation of stearic acid (18:0). They also observed an increase in α linolenic acid ( α -LNA) in the fat content of milk in these animals. However, there were no significant changes in rumenic acid (RA) concentrations in the milk fat, likely because the mammary gland continues to perform Δ 9-desaturation of vaccenic acid (VA).

**Figure 2:**
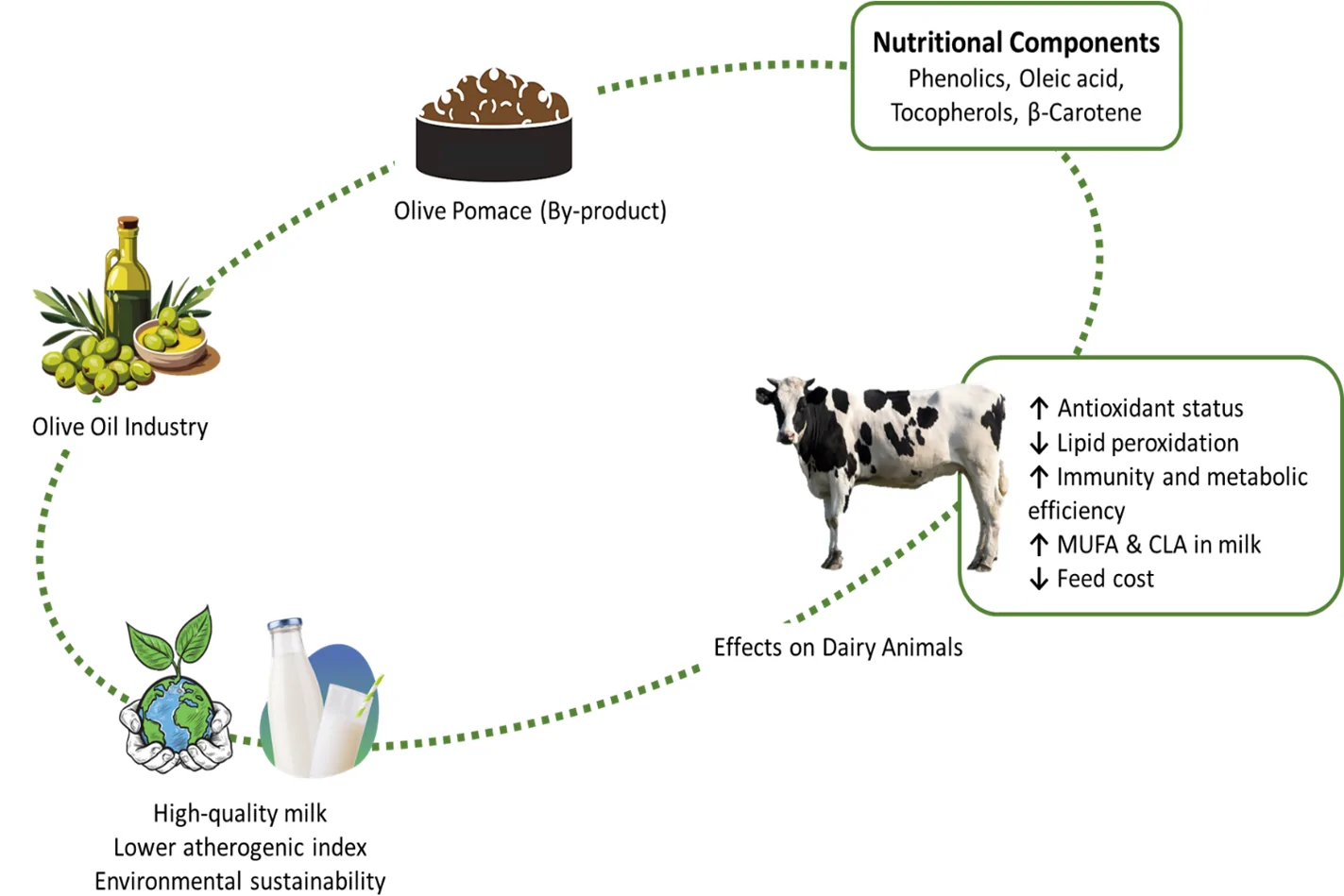
Schematic illustration showing how phenolics, tocopherols, and oleic acid act synergistically to enhance milk composition, antioxidant status, and environmental sustainability within a circular feed–health–productivity loop.

Adding dried olive pomace (DOP) to the diet did not change the general composition of the milk, but it did affect the protein content and the amount of urea in the milk, which remained within the ideal range of 23–35 mg/100 mL (Castellani et al., 2017). According to the results, milk somatic cell count did not vary; only the palmitic acid decreased. When cows were given olive pomace as a supplement, it resulted in higher levels of stearic acid, trans isomers of oleic acid (C18:1 trans-9 and C18:1 trans-11), oleic acid, rumenic acid, conjugated linoleic acid (CLA trans-10, cis-12), and long-chain fatty acids in their milk. Supplementation with olive pomace increases the MUFA mainly due to oleic acid, with a decrease in saturated fatty acids (SFAs) as well as in the atherogenic and thrombogenic indices of milk. Hence, it causes a rise in the milk content of conjugated linoleic acid (Zilio et al., 2014). The overall interactions between olive pomace bioactives, animal health, and milk quality are illustrated in Fig. 2, highlighting the integrated nutritional and sustainability benefits in dairy production systems.

Normally, milk yield is not affected by dietary DOP supplementation, as observed by researchers who found that dietary inclusion (about 15 % of DM) of olive pomace (dried stoned) did not improve milk production (Terramoccia et al., 2013). Although most studies have reported neutral or beneficial effects of olive pomace inclusion on milk yield and composition, the overall findings remain heterogeneous. These inconsistencies may reflect differences in the type of pomace (crude, ensiled, dried, or stoned), extraction method, inclusion rate, and baseline nutritional status of the animals. For instance, studies using dried or destoned pomace (Castellani et al., 2017; Zilio et al., 2014) generally found improved milk fatty acid profiles without affecting yield, while others using crude or high-moisture pomace observed reductions in nutrient digestibility and feed efficiency (Owaimer et al., 2004; García et al., 2003). Such contrasts highlight the methodological variability across studies and emphasize the need for standardized experimental protocols and better characterization of olive pomace composition prior to inclusion in ruminant diets.

Moreover, limited data exist on long-term metabolic and immunological responses to olive pomace feeding in dairy animals. Most available studies are short term and focus primarily on production performance, with fewer examining oxidative or inflammatory biomarkers. Addressing these gaps could help determine whether the observed nutritional benefits translate into measurable health and welfare improvements.

Adding olive pomace to the diet helps boost the development of γ lactones in milk, thanks to the oleic acid provided by olive pomace. Oleic acid acts as a precursor to 10-hydroxy stearic acid in the rumen, which is then converted into γ -12:0 in the mammary gland through β oxidation (Jenkins et al., 2006). Olive pomace supplementation in diets with high levels of oleic acid may be responsible for decreasing the δ lactones in milk. It is predicted that ruminal acetate, a substance that serves as a precursor to lactones in the mammary gland, is suppressed by dietary unsaturated fatty acids (Griinari et al., 1998) by limiting the digestion of fibres. Despite the numerous studies linking olive pomace inclusion to improved milk fatty acid composition, the underlying biochemical mechanisms remain only partially understood. While many authors attribute these effects to the high oleic acid content and the presence of polyphenols that modulate ruminal biohydrogenation (Pallara et al., 2014; Molina-Alcaide et al., 2010), other data suggest that these bioactives can also interfere with microbial enzyme activity, thereby reducing fibre digestibility and volatile fatty acid (VFA) production (Tufarelli et al., 2013; García et al., 2003).

Furthermore, most in vitro studies have been conducted under simplified conditions that may not accurately represent the dynamic interactions within the rumen ecosystem. This limits the extrapolation of their findings to real feeding systems. For example, changes in the abundance of specific microbial taxa such as *Butyrivibrio proteoclasticus* and *Megasphaera elsdenii* have been proposed (Vasta et al., 2010), but few in vivo studies have validated these shifts through metagenomic or metabolomic profiling. Future work integrating rumen microbial sequencing and lipidomics could clarify the causal links between olive pomace bioactives, rumen metabolism, and milk lipid quality.

**Figure 3:**
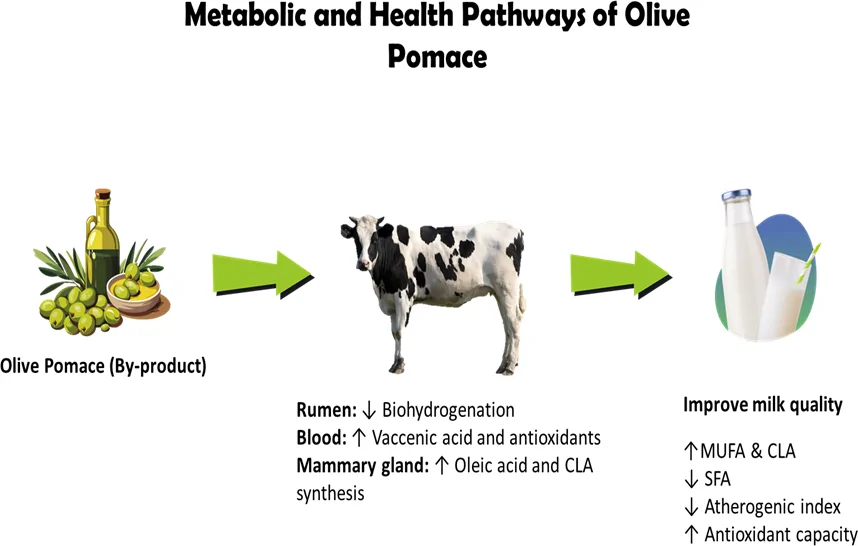
Proposed metabolic and health pathways linking olive pomace supplementation to improved milk fatty acid composition. The diagram illustrates the flow of bioactive compounds from feed ingestion to ruminal modulation, systemic antioxidant effects, and mammary lipid metabolism, resulting in enhanced milk MUFA and CLA content and reduced SFA levels.

Adding DOP to the diet affected milk protein content, an unexpected result that is inconsistent with previous studies where feeding olive pomace to ewes (Abbeddou et al., 2011) and cows (Zilio et al., 2014) showed no change in protein content. Even though the diets were balanced in energy and protein, cows in the experimental group (EG) were fed less forage than those in the control group (CG), and the difference in the ratio of forage to concentrate might have contributed to the higher protein yield (Jenkins and McGuire, 2006). Furthermore, Pallara et al. (2014) conducted an in vitro study and found that olive pomace inclusion stimulated the production of volatile fatty acids (VFAs) in rumen. Researchers have shown that olive pomace integration can modify ruminal microflora activity and the protein composition of milk. Nudda et al. (2005) showed that DOP inclusion has a promising effect on fatty acid levels in milk. The proposed metabolic pathway through which olive pomace bioactives influence ruminal metabolism and the milk lipid profile is presented in Fig. 3.

Beyond its effects on milk composition, olive pomace may also influence the metabolic and oxidative status of dairy animals. The high content of phenolic compounds, tocopherols, and unsaturated fatty acids provides strong antioxidant potential, which could protect cellular membranes and mammary tissues against lipid peroxidation (Benincasa et al., 2021; Mohammadabadi et al., 2025). However, most available studies have evaluated these effects indirectly through changes in the milk fatty acid profile or antioxidant activity in milk, while direct assessments of systemic oxidative or immune biomarkers in animals remain scarce.

Furthermore, discrepancies exist regarding the magnitude of these health-related effects, likely due to variations in pomace composition, storage stability, and drying temperature, which can markedly affect phenolic retention and bioavailability. Few studies have measured enzymatic antioxidants such as glutathione peroxidase or catalase activity in plasma, making it difficult to determine whether the antioxidant benefits of olive pomace extend beyond the mammary gland level (Benincasa et al., 2021; Mohammadabadi et al., 2025). Consequently, integrating physiological and immunological markers in future experiments would help clarify whether the nutritional inclusion of olive pomace contributes to overall animal health and resilience, not only to milk compositional improvements.

Several studies have looked into how olive pomace or tannins affect the ruminal microbial community, especially *Butyrivibrio proteoclasticus* and the *Butyrivibrio* genus, which are responsible for hydrogenating unsaturated fatty acids (UFAs) and converting C18:1 to C18:0. Olive pomace has been found to reduce the activity of these microbes, slowing down the hydrogenation process of oleic and linoleic acid intermediates (Vasta et al., 2010). The presence of vaccenic acid, trans isomers of oleic acid, and rumenic acid in milk is regulated by the enzyme stearoyl coenzyme A desaturase in the mammary tissue. While the partial BH of linoleic acid in the rumen contributes a small amount of CLA to the milk (Chilliard and Ferlay, 2004), most of the rumenic acid secreted in the milk (about 60 %) is produced by the mammary gland starting from vaccenic acid (Mosley et al., 2006). When olive pomace is added to ruminant diets, it can cause different changes, including alterations in ruminal fermentation, VFA distribution, and biohydrogenation. The specific effects depend largely on how much is used and how it is incorporated into the diet. Olive pomace is an inexpensive source of energy and fibre, which can help improve the percentage of fat in milk and enhance the quality of fat in animal products (Mohammadabadi et al., 2025; Paié-Ribeiro et al., 2025).

## 4 Effects of olive pomace on nutrient digestibility

Limited data are available on the effects of olive pomace in animal feed on nutrient digestibility of other dietary nutrients. Olive pomace generally has less digestibility and energy metabolism, which could be attributed to high levels of tannins, high amounts of lignin, and closeness of proteins with lignocellulose in them (Sadeghi et al., 2009). It has been reported previously that dietary inclusion of olive pomace decreased the digestibility of NDF, DM, and ADF by up to 10 %–30 % (Al-Jasim et al., 1997). It was further verified through a sacco experiment that neutral detergent fibre in the olive pomace takes extra time to degrade as compared to other plants' by-products (Abbeddou et al., 2011). Additionally, the Maillard reaction due to heat production during the extraction process and reduced availability of nitrogen in the rumen may exert negative effects on ruminal microbes and could be responsible for the decrease in crude protein digestibility (Obeidat and Kridli, 2021). In the literature, antagonistic effects of olive pomace on other ingredients were reported to be responsible for decreased digestibility of nutrients in lambs fed 34 % olive pomace compared to those fed the control diet (Abbeddou et al., 2011). For instance, decreased nitrogen balance was observed in lambs fed 12 % olive pomace in their diet in a different study (Owaimer et al., 2004). Various studies also reported a decrease in the digestibility of other nutrients, including DM, NDF, and ADF, in diets in which olive pomace was included in the ration (García et al., 2003; Yáñez-Ruiz and Molina-Alcaide, 2007; Tufarelli et al., 2013). In contrast, a positive effect on nitrogen balance, without affecting nitrogen intake and nitrogen excretion, in diets containing 15 % olive pomace has also been observed (Obeidat, 2019). Moreover, no difference in the digestibility of NDF, OM, DM, and CP has been observed with the addition of olive pomace in the diet (Awawdeh and Obeidat, 2013). Similarly, another study reported that the addition of olive pomace in sheep feed enhanced the digestibility of NDF, OM, DM, and CP (Sadeghi et al., 2009). Interestingly, in another study, although increasing the olive pomace level in the diet decreased digestibility, feed intake was increased, which resulted in improved balance even at the maximum inclusion level of olive pomace in the beef cattle feed (Estaún et al., 2014). These contradictions in the literature may be attributed to the limited number of studies available on the matter, which vary in the inclusion rate of olive pomace, the method of olive oil extraction, and experimental approaches (Obeidat and Kridli, 2021).

The inconsistent findings across studies investigating nutrient digestibility suggest that several methodological and compositional factors may have confounded the results. For instance, the variation in the chemical composition of olive pomace due to the extraction method (two- or three-phase systems), degree of destoning, and drying temperature can substantially affect fibre structure and phenolic content, thereby altering ruminal degradability (Obeidat and Kridli, 2021). High levels of lignin and condensed tannins are known to bind proteins and inhibit fibrolytic enzymes, which may explain the lower digestibility of neutral detergent fibre (NDF) and crude protein reported by some authors (Al-Jasim et al., 1997; Abbeddou et al., 2011).

In contrast, studies that used properly processed and partially destoned olive pomace (Sadeghi et al., 2009; Awawdeh and Obeidat, 2013) have shown neutral or even improved digestibility, indicating that technological treatment plays a critical role in determining nutritional value. This variability highlights the importance of characterizing the physicochemical properties of pomace before dietary inclusion.

Another major limitation in the available literature is the short experimental duration and the absence of kinetic digestion data. Most studies relied on in vitro or in sacco assays that do not fully reflect in vivo ruminal conditions. Standardized long-term trials incorporating both digestibility and fermentation kinetics could better quantify the actual nutritive contribution of olive pomace in dairy animal diets (Awawdeh and Obeidat, 2013).

## 5 Economics of olive pomace

Using olive pomace in animal feed is a promising idea, as it can significantly reduce feed costs, with some studies indicating potential savings of up to 75 %. But it needs to be processed correctly to eradicate problems associated with high fibre content and possible taste concerns (Sevillano et al., 2025; Schú et al., 2025). Adding it can increase the quality of meat and milk, which can lead to economic gains through higher-value goods. Giving dairy cows olive pomace can save money on feed and possibly make milk products worth more. Recent studies indicate that it can save feed costs by as much as 6.5 % and is a cheap way to get rid of waste (Castellani et al., 2017; Schú et al., 2025). It can also improve the quality of milk, which could lead to a better price. However, at greater inclusion rates, it may have little or no influence on milk yield (Molina-Alcaide and Yáñez-Ruiz, 2008; Castellani et al., 2017). Using olive pomace in animal feed is an innovative and long-lasting way to reduce waste. It might also lower the expenses of feeding animals, which would make waste biomass more valuable.

There are a number of factors that affect the economic viability of utilizing OC in animal feed, such as how close olive oil mills are to pig-producing units. The Trás-os-Montes region has many Portuguese mills and a lot of these pig breeds. Because olive oil manufacturing and animal farming are close to each other, it is easy to use OC in animal feed. Pomace must be dried, which is energy-intensive, making it a poor animal feed. This factor is a big problem since compound feed factories do not use pomace that has a lot of water in it. Even though energy consumption can be an issue, the next step in removing leftover fat using chemical solvents already involves drying the pomace (Paié-Ribeiro et al., 2024). Consequently, rather than adhering to this industrial procedure, its direct application in animal feed may provide a more economically beneficial option (Paié-Ribeiro et al., 2025).

Although most reports emphasize the economic advantages of olive pomace as a low-cost feed ingredient, a more critical assessment reveals several practical and logistical constraints that may limit its large-scale adoption. Its economic benefit is highly dependent on regional availability, transportation distance, and the need for drying or ensiling, which can significantly increase processing costs (Abbeddou et al., 2011; Owaimer et al., 2004). Additionally, the seasonal nature of olive oil production creates fluctuations in supply and quality, making year-round utilization challenging. Another issue rarely addressed in the literature is the potential trade-off between cost savings and nutrient efficiency. While inclusion of olive pomace may reduce feed costs, excessive levels can impair digestibility and animal performance, thereby diminishing overall profitability (Chiofalo et al., 2004). Moreover, the lack of detailed lifecycle or cost–benefit analyses limits our understanding of its true economic sustainability when all inputs, such as energy for drying and environmental management, are accounted for.

Future research should therefore integrate techno-economic and environmental modelling to quantify the net economic and ecological benefits of olive pomace use under different production scenarios. Such an approach would provide a more holistic framework aligning with circular economy principles and sustainable livestock production.

## 6 Nutritional and digestive limitations

Olive pomace has several big problems when it comes to feeding animals. For example, it contains a lot of fibre, which can make it harder for monogastric animals, including poultry, to digest and grow. Olive pomace also has tannins and polyphenols, which make it less tasty and less nutritious (Obeidat et al., 2019). Other issues include high fat content, low protein content that needs to be added carefully, the possibility of oxidation and poor nutritional value after drying, and differing quality depending on how it is processed (Obeidat et al., 2019). It also contains a high percentage of crude fibre (33 %–42.60 %). Moreover, its protein content is not very high (6.6 %–9.9 %), which means it may not work well as a key source of protein. However, it can be an excellent source of energy, but because it has a lot of fat (10 %–30 %), it should only be eaten in small amounts (about 10 % of animals' overall diet) (Molina-Alcaide and Yáñez-Ruiz, 2008).

Olive pomace contains some anti-nutritional factors like tannins and polyphenols, which can make food taste bad and make it difficult to digest, and it can also inhibit the activity of vital microbes in rumen (Al-Harthi, 2016). On the other hand, phytic acid is also present in olive pomace and can stop the body from absorbing nutrients. In the same context, there are some nutritional problems associated with processing olive waste (Nagarajaiah and Prakash, 2016). Drying is the standard method for preserving food, but it can cause oxidation of good fatty acids, decreasing the nutritive value of the food. The quality of olive pomace can vary greatly depending on factors such as the kind of olive, the grinding technique (two stage vs. three stage), and whether the pits have been removed. Nevertheless, fresh olive remains contain a lot of water and do not last long; therefore, they need to be preserved, which may alter their quality. Therefore, its effectiveness can vary greatly depending on the amount used in the diet and the specific type (Molina-Alcaide et al., 2003).

Despite the recognized nutritional potential of olive pomace, several limitations remain unresolved, particularly concerning its polyphenolic composition and fibre content. High contents of lignin and tannin can impair enzyme activity in the rumen and microbial attachment and can reduce both nutrient digestibility and nitrogen efficiency (Abbeddou et al., 2011; Owaimer et al., 2004). However, such effects are highly variable depending on the source and pretreatment of olive pomace. For example, mechanical destoning and mild drying have been shown to improve digestibility by disrupting lignocellulosic structures (Sadeghi et al., 2009), whereas high-temperature processing may increase oxidative polymerization and reduce phenolic bioactivity and nutrient availability (Estaún et al., 2014).

Another critical gap is the insufficient understanding of how olive pomace interacts with rumen microbial communities. Although some in vitro studies have reported selective inhibition of cellulolytic and proteolytic bacteria, comprehensive in vivo evaluations using high-throughput sequencing are still lacking (Abbeddou et al., 2011; Owaimer et al., 2004). Therefore, it remains difficult to determine optimal levels that balance antimicrobial impacts with efficiency of fibre degradation. Addressing these challenges requires a combined approach integrating feed technology (ensiling, enzymatic treatment, fermentation) with omics-based microbial analyses to improve both the functional and the nutritional value of olive pomace in feeding systems of ruminants (Abbeddou et al., 2011; Owaimer et al., 2004).

## 7 Conclusions

Olive pomace represents a promising, cost-effective, and environmentally sustainable by-product that can serve as an alternative feed ingredient for dairy animals. Its rich composition of phenolics, unsaturated fatty acids, and antioxidants provides multiple functional benefits, including improved milk quality, enhanced oxidative stability, and potential health-promoting effects. However, the current literature still lacks a consistent framework explaining the mechanisms behind these effects. Most studies focus on production and composition parameters, while fewer address metabolic, microbial, and immunological responses. The variability in pomace source, processing methods, and inclusion levels complicates direct comparisons and hinders the establishment of standardized feeding recommendations. Overall, olive pomace holds great potential for enhancing both the nutritional and the environmental sustainability of dairy systems, but its optimal use requires a deeper mechanistic understanding, technological optimization, and validation under diverse production conditions.

## Data Availability

All the data generated during this study are included in this published article.

## Future research directions

Future studies should focus on several key areas to consolidate the role of olive pomace in sustainable dairy nutrition:

– *Mechanistic insights*. Explore the molecular and cellular mechanisms through which olive pomace polyphenols and lipids influence rumen microbial ecology, metabolic efficiency, and immune function.

– *Technological innovation*. Develop and evaluate new processing strategies, such as enzymatic pretreatment, microbial fermentation, and co-ensiling, to enhance the bioavailability of nutrients and minimize antinutritive factors.

– *Long-term and integrative trials*. Conduct extended in vivo studies combining production performance with biochemical, immunological, and metabolomic profiling to establish causal relationships and ensure animal welfare benefits.

– *Sustainability and circular economy modelling*. Apply lifecycle and techno-economic analyses to quantify the net environmental and financial gains of olive pomace utilization in different production systems.

By integrating these multidisciplinary approaches, future research can move beyond descriptive findings toward a more predictive, systems-based understanding of how olive pomace contributes to animal health, product quality, and agricultural sustainability.
